# Supplementary material for: Delta-Radiomics Predicts Response to First-Line Oxaliplatin-Based Chemotherapy in Colorectal Cancer Patients with Liver Metastases
Source: Cancers (Basel). 2022 Jan 4;14(1):241. doi: 10.3390/cancers14010241 (PMC8750408; doi:10.3390/cancers14010241)
Supplement: Supplementary file 1 [file cancers-14-00241-s001.zip › cancers-1531682-supplementary.pdf]

cancers

MDPI

Table S1. Reporting Guidelines: from Image processing to features calculation steps.

Area	Topic	Description
Patient	Region of interest	Liver metastases
Acquisition	Acquisition protocol	Different acquisition protocols (2 centers)
	Scanner type	Center A: Siemens, Somaton Definition/ Sensation 64<br>Center B: Siemens Somaton Definition FLASH
	Imaging modality	TC
	Scan duration	Approximately 10/15 minutes
Image registration	Registration method	NONE
Data conversion	Not applicable	NONE
Post-acquisition processing	Anti-aliasing	NONE
	Non-uniformity correction	NONE
	Intensity normalization	NONE
Segmentation	Method	Manual segmentation performed on portal-phase CT scan
	Conversion to mask	NIFTI
Image Interpolation	Interpolation method	NONE
Mask Interpolation	Interpolation method	NONE
Re-segmentation	Method	Between 1st and 99th percentile
Discretization	Method	Fixed bin number (32 bin)
Image transformation	Image filter	NONE
Image biomarker computation	Biomarker set	Shape-based, First-order statistics, GLCM, GLRLM, GLSZM, NGTDM, GLDM
	IBSI compliance	Yes
	Software availability	Pyradiomics
Image biomarker computation - texture parameters	Texture matrix aggregation	2D averaged
	Distance weighting	No weighting
	CM symmetry	Symmetric co-occurrence matrices
	CM distance	1
List of features	First-order statistics	Energy<br>total energy

	entropy<br>minimum<br>10th<br>90th<br>Maximum<br>Mean<br>Median<br>interquartile range<br>range<br>mean absolute deviation<br>robust mean absolute deviation<br>root mean squared<br>skewness<br>kurtosis<br>variance<br>uniformity
Shape-based	Elongation<br>Flatness<br>Least Axis Length<br>Major Axis Length<br>Maximum 2D Diameter Column<br>Maximum 2D Diameter Row<br>Maximum 2D Diameter Slice<br>Maximum3DDiameter<br>Mesh Volume<br>Minor Axis Length<br>Sphericity<br>Surface Area<br>Surface Volume Ratio<br>Voxel Volume
GLCM	Autocorrelation<br>Cluster Prominence<br>Cluster Shade<br>Cluster Tendency<br>Contrast<br>Correlation<br>Difference Average<br>Difference Entropy<br>Difference Variance<br>Inverse Difference<br>Normalized Inverse Difference<br>Inverse Difference Moment<br>Normalized Inverse Difference Moment<br>Informational Measure of Correlation 1<br>Informational Measure of Correlation 2<br>Inverse Variance<br>Joint Average<br>Joint Energy<br>Joint Entropy<br>Maximal Correlation Coefficient<br>Maximum Probability<br>Sum Average<br>Sum Entropy<br>Sum Squares
GLRLM	Gray Level Non-Uniformity<br>Gray Level Non-Uniformity Normalized<br>Gray Level Variance<br>High Gray Level Run Emphasis<br>Long Run Emphasis

	Long Run High Gray Level Emphasis<br>Long Run Low Gray Level Emphasis<br>Low Gray Level Run Emphasis<br>Run Entropy<br>Run Length Non-Uniformity<br>Run Length Non-Uniformity Normalized<br>Run Percentage<br>Run Variance<br>Short Run Emphasis
GLSZM	Gray Level Non-Uniformity<br>Gray Level Non-Uniformity Normalized<br>Gray Level Variance<br>High Gray Level Zone Emphasis<br>Large Area Emphasis<br>Large Area High Gray Level Emphasis<br>Large Area Low Gray Level Emphasis<br>Low Gray Level Zone Emphasis<br>Size Zone Non-Uniformity<br>Size Zone Non-Uniformity Normalized<br>Small Area Emphasis<br>Small Area High Gray Level Emphasis<br>Small Area Low Gray Level Emphasis<br>Zone Entropy
NGTDM	Busyness<br>Coarseness<br>Complexity<br>Contrast<br>Strength
GLDM	Dependence Entropy<br>Dependence Non-Uniformity<br>Dependence Non-Uniformity Normalized<br>Dependence Variance<br>Gray Level Non-Uniformity<br>Gray Level Variance<br>High Gray Level Emphasis<br>Large Dependence Emphasis<br>Large Dependence High Gray Level Emphasis<br>Large Dependence Low Gray Level Emphasis<br>Low Gray Level Emphasis<br>Small Dependence Emphasis<br>Small Dependence High Gray Level Emphasis<br>Small Dependence Low Gray Level Emphasis

Table S2. Performances of other ML techniques on both training and validation sets.

	**Train**					**Validation**				
	**ACC % (95% CI)**	**SE % (95% CI)**	**SP % (95% CI)**	**PPV % (95% CI)**	**NPV % (95% CI)**	**ACC % (95% CI)**	**SE % (95% CI)**	**SP % (95% CI)**	**PPV % (95% CI)**	**NPV % (95% CI)**
Decision Tree	97 (89-100)	99 (94-99)	94 (85-98)	95 (89-98)	99 (91-100)	86 (81-92)	85 (68-95)	92 (78-98)	90 (76-96)	87 (75-94)
LR Step-wise binomial	81.5 (77-84)	79 (73-81)	84 (73-85)	86 (72-90)	76 (73-80)	63 (55-69)	61 (50-65)	65 (51-70)	61 (50-65)	65 (50-65)
LR Step-wise poisson	78.5 (73-81)	79 (73-81)	78 (72-82)	81 (74-83)	74 (71-80)	77.5 (69-84)	82 (70-85)	73 (68-79)	73 (69-79)	82 (69-85)
SVM Linear	79	84	73.5	80	61	67.5	67.5	67.5	62.5	67.5

	(71-83)	(72-85)	(71-81)	(71-84)	(56-67)	(58-75)	(58-71)	(61-75)	(55-68)	(61-75)
Random Forest	93 (88-96)	94 (84-96)	92 (83-94)	94 (86-97)	92 (81-99)	75 (67-81)	64 (55-71)	86 (71-89)	80 (71-89)	72 (65-83)

LR: logistic regression, ACC: accuracy, SE: sensitivity, SP: specificity, PPV: positive predictive value, NPV: negative predictive value.

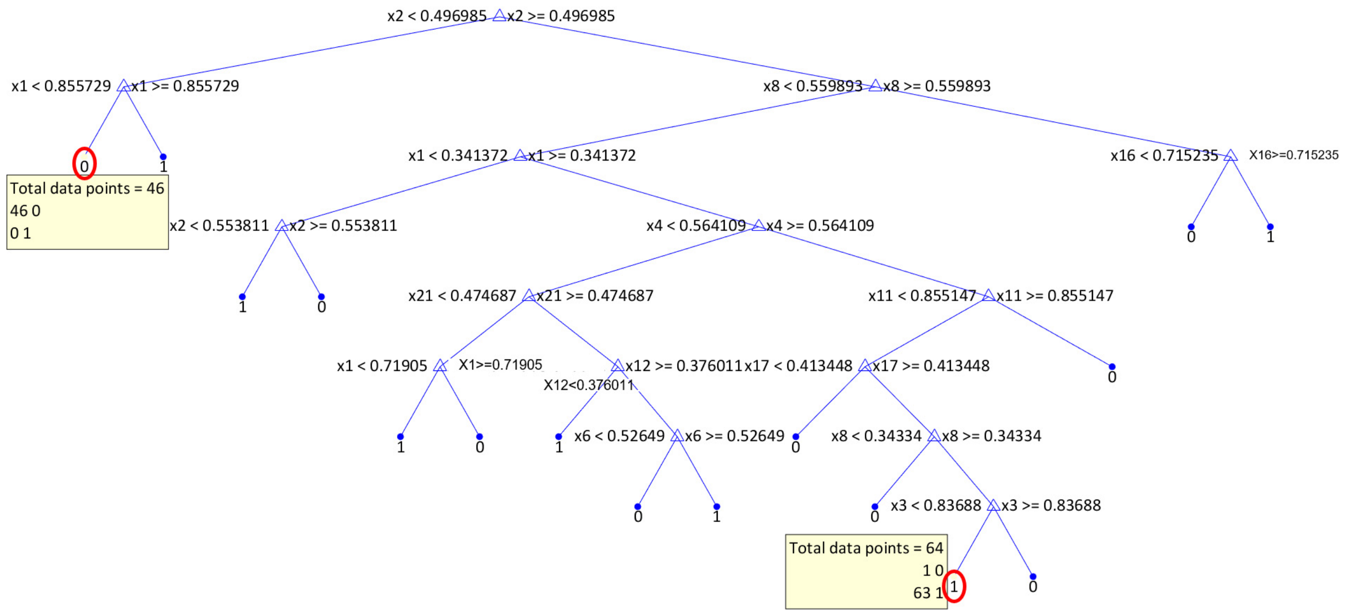

Figure S1. Trained Decision Tree containing the 11 selected features: shape Sphericity (named x1 in Figure 2), shape Surface Volume Ratio (x2), GLCM Contrast (x3), GLCM Difference Average (x4), GLCM Difference Variance (x6), GLCM Maximum Probability (x8), GLDM Small Dependence Low Gray Level Emphasis (x11), GLRLM Run Length Non Uniformity Normalized (x12), GLSZM Size Zone Non Uniformity Normalized (x16), GLSZM Small Area Emphasis (x17), and NGTDM Complexity (x21). 46 non responder lesions in the training set (27%) were correctly classified using only 2 variables (shape sphericity and shape surface volume ratio). In particular, if the value of both features was lower than the threshold computed by the model, these lmCRC were classified as non-responder. Conversely, to classify a lesion as R+ more features were needed, as it is visible from the last leaf of the DT, that classifies 63 lesions as R+.

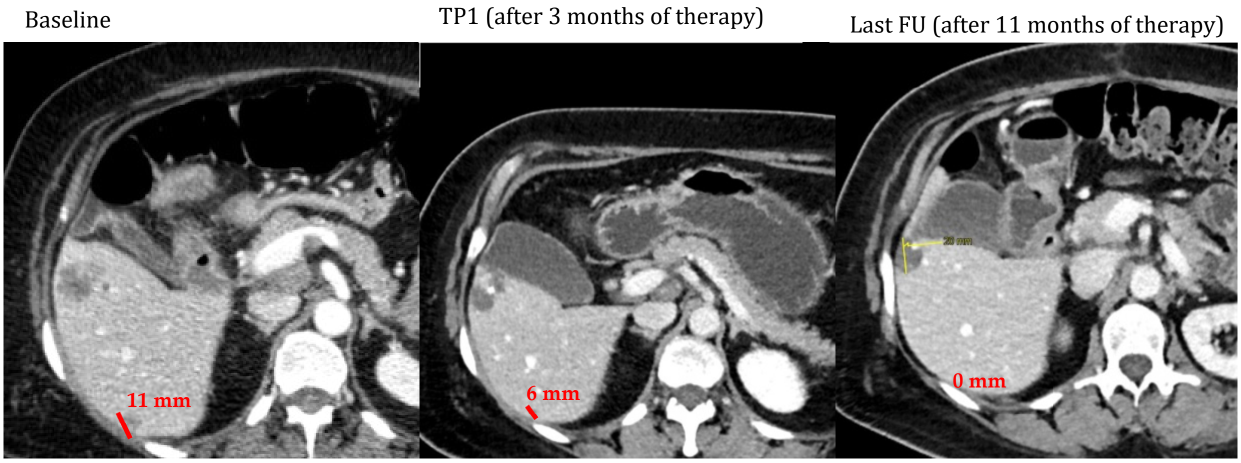

Figure S2. Lesion 1 in patient 1010 at baseline, TP1 and last FU during first-line chemotherapy. Lesion 1 was a good responder misclassified as R- by the algorithm (CR at 11 months). Probably, small size could have hindered a correct segmentation in this case.

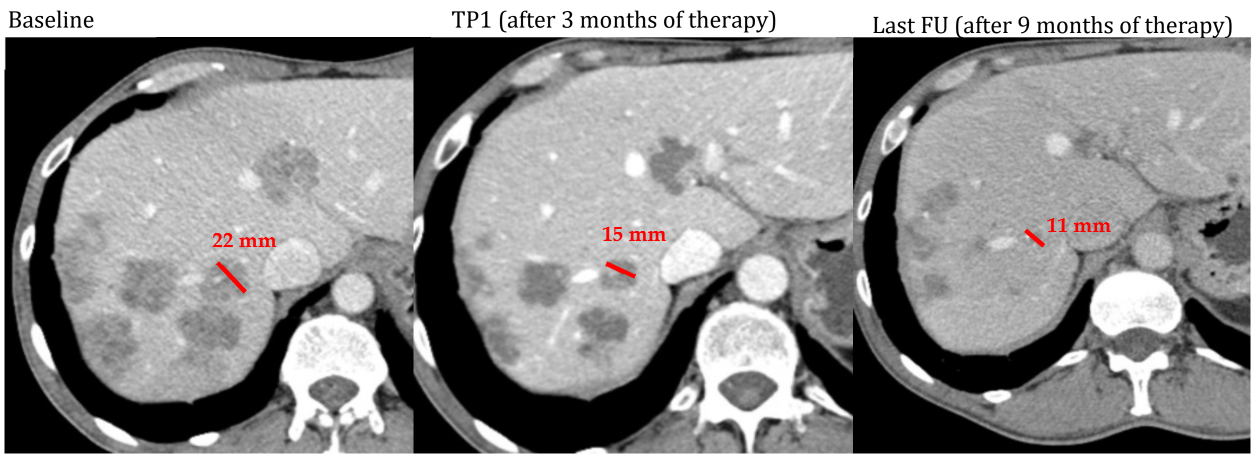

Figure S3. Lesion 4 in patient 1016 at baseline, TP1 and last FU during first-line chemotherapy. This metastasis was a good responder misclassified as R- by the algorithm (PR at 9 months).

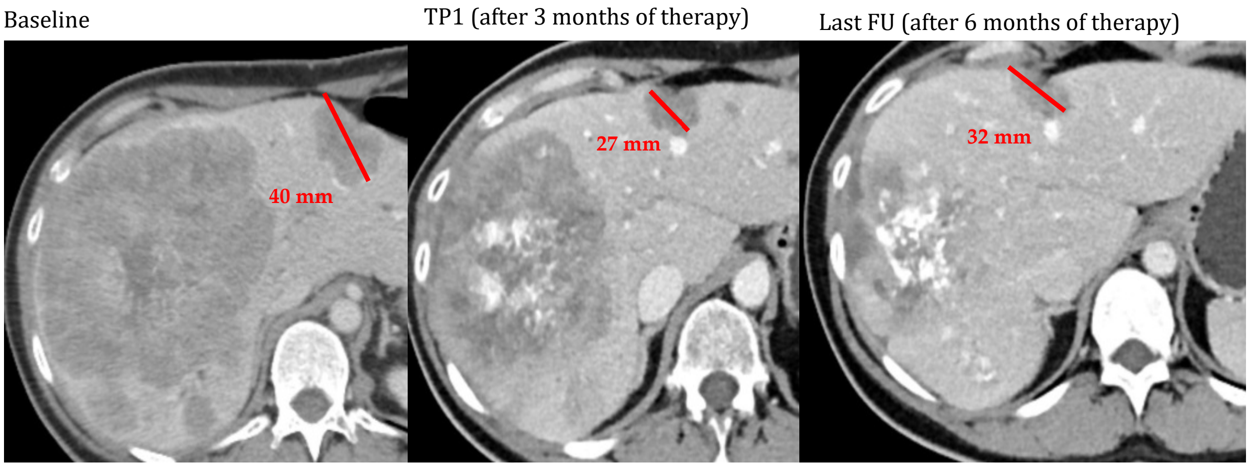

(**a**)

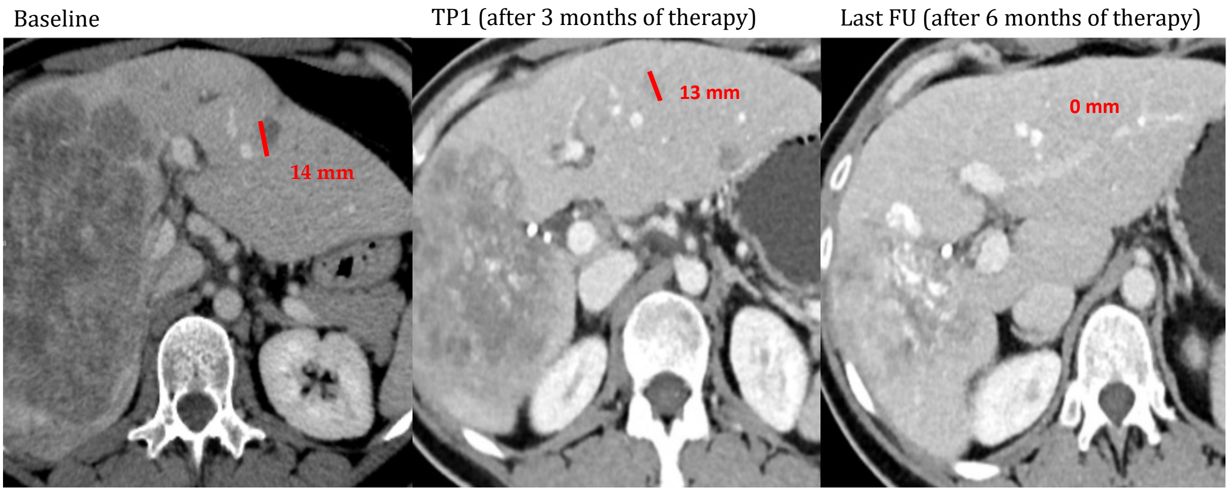

(**b**)

Figure S4. (**a**) Lesion 2 in patient 1017 at baseline, TP1 and last FU during first-line chemotherapy. Lesion 2 was a bad responder misclassified as R+ by the algorithm (PD at 6 months). (**b**) Lesion 4 in patient 1017 at baseline, TP1 and last FU during first-line chemotherapy. Lesion 7 was a good responder misclassified as R- by the algorithm (CR at 6 months).

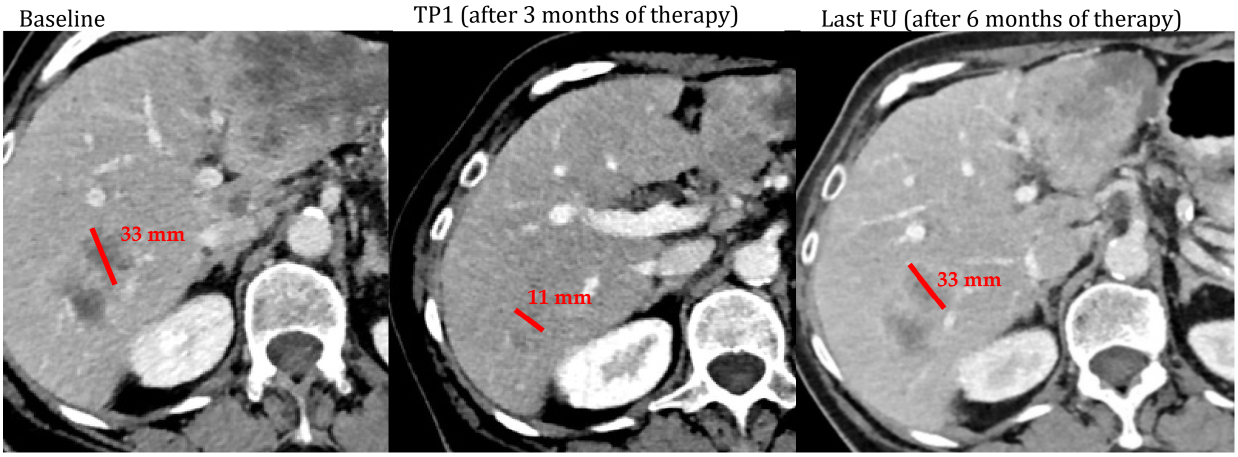

(**a**)

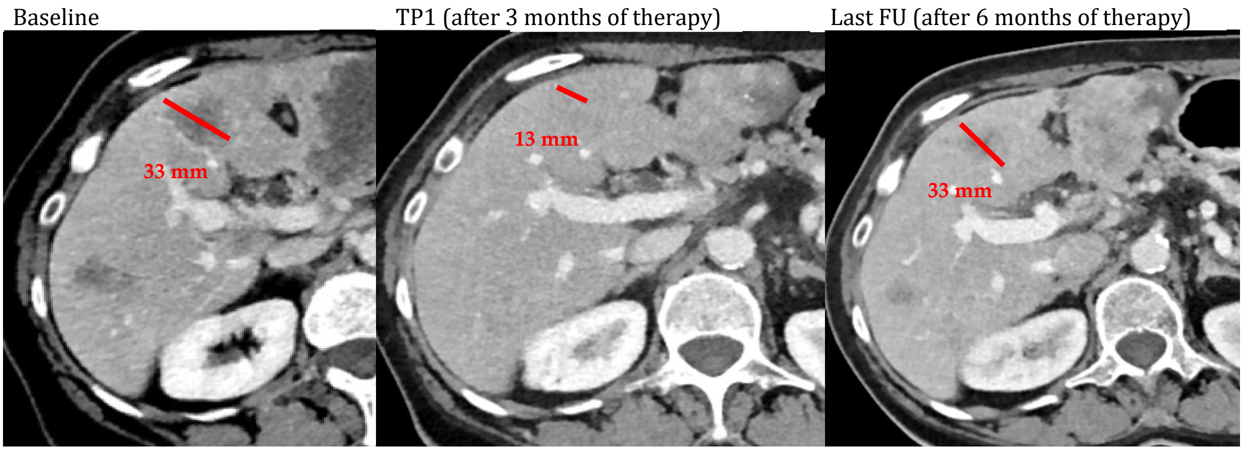

(**b**)

Figure S5. (**a**) Lesion 4 in patient 1010 at baseline, TP1 and last FU during first-line chemotherapy. Lesion 4 was a bad responder misclassified as R+ by the algorithm (PD at 6 months). To note that a PR was recorded at TP1. (**b**) Lesion 7 in patient 1010 at baseline, TP1 and last FU during first-line chemotherapy. Lesion 4 was a bad responder misclassified as R+ by the algorithm (PD at 6 months). To note that a PR was recorded at TP1. The lesion was almost indistinguishable from normal liver parenchyma at TP1, being a possible source for incorrect segmentation.
